# COUNTEN, an AI-Driven Tool for Rapid and Objective Structural Analyses of the Enteric Nervous System

**DOI:** 10.1523/ENEURO.0092-21.2021

**Published:** 2021-07-23

**Authors:** Yuta Kobayashi, Alicia Bukowski, Subhamoy Das, Cedric Espenel, Julieta Gomez-Frittelli, Narayani Wagle, Shriya Bakshi, Monalee Saha, Julia A. Kaltschmidt, Archana Venkataraman, Subhash Kulkarni

**Affiliations:** 1 Department of Computer Science, Whiting School of Engineering, Johns Hopkins University, Baltimore, MD 21218; 2 Center for Neurogastroenterology, Division of Gastroenterology and Hepatology, Department of Medicine, Johns Hopkins University, Baltimore, MD 21205; 3 Department of Neurosurgery, Stanford University School of Medicine, Stanford, CA 94305; 4 Cell Sciences Imaging Facility, Stanford University School of Medicine, Stanford, CA 94305; 5 Department of Chemical Engineering, Stanford University, Stanford, CA 94305; 6 Wu Tsai Neurosciences Institute, Stanford University, Stanford, CA 94305; 7 Department of Electrical and Computer Engineering, Whiting School of Engineering, Johns Hopkins University, Baltimore, MD 21218

**Keywords:** artificial intelligence, computational, enteric nervous system, myenteric plexus, open-source tool, organization

## Abstract

The enteric nervous system (ENS) consists of an interconnected meshwork of neurons and glia residing within the wall of the gastrointestinal (GI) tract. While healthy GI function is associated with healthy ENS structure, defined by the normal distribution of neurons within ganglia of the ENS, a comprehensive understanding of normal neuronal distribution and ganglionic organization in the ENS is lacking. Current methodologies for manual enumeration of neurons parse only limited tissue regions and are prone to error, subjective bias, and peer-to-peer discordance. There is accordingly a need for robust, and objective tools that can capture and quantify enteric neurons within multiple ganglia over large areas of tissue. Here, we report on the development of an AI-driven tool, COUNTEN (COUNTing Enteric Neurons), which is capable of accurately identifying and enumerating immunolabeled enteric neurons, and objectively clustering them into ganglia. We tested and found that COUNTEN matches trained humans in its accuracy while taking a fraction of the time to complete the analyses. Finally, we use COUNTEN’s accuracy and speed to identify and cluster thousands of ileal myenteric neurons into hundreds of ganglia to compute metrics that help define the normal structure of the ileal myenteric plexus. To facilitate reproducible, robust, and objective measures of ENS structure across mouse models, experiments, and institutions, COUNTEN is freely and openly available to all researchers.

## Significance Statement

COUNTEN (COUNTing Enteric Neurons) is the first open-source AI-driven tool that performs automated, rapid, and objective enumeration and clustering of murine enteric neurons.

## Introduction

Gastrointestinal (GI) motility is regulated by the enteric nervous system (ENS; Spencer and Hu, 2020). The majority of the neurons and glia of the ENS are contained within the myenteric plexus, where they are clustered in interconnected ganglia of various sizes (Sternini, 1988). Alterations in ENS structure, gauged by parameters such as neuron density or number of neurons per ganglia, are associated with GI dysmotility (Rao and Gershon, 2018). While ENS structure is thus relevant to health and disease, methods by which it can be comprehensively assessed are limited. Currently there are no objective methods to capture and quantify enteric neurons over large tissue areas or within multiple ganglia. The challenge of objectively quantifying ENS structure at both the neuronal and ganglionic level is particularly acute. While some studies have calculated aggregate myenteric neuronal densities (Gabella, 1987; Santer and Baker, 1988; Kunze and Furness, 1999), they have addressed the ganglionic organization of these neurons (defined here as the number of neurons per individual ganglia, or number of ganglia per area of tissue) in other limited ways.). Recent studies have computed average ganglia size (Gianino et al., 2003; Becker et al., 2018); however, because of the laborious process of manually counting and clustering neurons, these studies have analyzed only isolated tissue areas, thus precluding more comprehensive understanding of the structure of the adult myenteric plexus. In addition, since myenteric ganglia show a diversity of shapes and sizes, and exhibit varying degrees of proximity from each other, manual enumeration and clustering of neurons into ganglia is prone to error, subjective bias, and peer-to-peer discordance. Hence, there is a critical need for objective and rapid tools and methods for standardized enumeration and classification of myenteric neurons into ganglia over large tissue areas to build a comprehensive understanding of ENS structure.

In this report, we present COUNTEN (COUNTing Enteric Neurons), the first automated, open-source software that uses computer-vision and image-processing methods for high-throughput analysis of widefield microscopy images to reliably identify, enumerate, and cluster myenteric plexus neurons in a rapid and objective manner.

## Materials and Methods

### Animals

All animal experiments were conducted in accordance with the protocols that were approved by the Animal Care and Use Committee of Johns Hopkins University in accordance with the guidelines provided by the National Institutes of Health. Nine-week-old littermate male mice from the C57BL/6 (Charles River) background, which were housed in the same cage, were used for the experiment.

### Tissue isolation

Mice were anesthetized with isoflurane and killed by cervical dislocation. An abdominal incision was made to perform a laparotomy and isolate intestines that were gently pulled out and placed in a clean Petri dish containing sterile ice-cold Opti-MEM solution. The intestinal contents were flushed using ice-cold sterile PBS after which the terminal ileum, defined as the last 5 cm of small intestinal tissue before the cecum, was dissected out. The longitudinal muscle containing myenteric plexus (LM-MP) tissue from the terminal ileum was peeled out with a sterile clean cotton swab, cleaned in sterile ice-cold OptiMEM, flattened on a dish and fixed with freshly prepared ice-cold 4% paraformaldehyde solution for 30 min. The tissue was washed in sterile ice-cold PBS and used for immunostaining.

### Immunostaining

Fixed LM-MP tissues were incubated at room temperature (RT) while shaking in blocking permeabilization buffer (BPB; 5% normal goat serum, 0.1% Triton X-100 in sterile PBS), after which tissues were washed in sterile PBS and incubated overnight with rabbit anti-HuC/D primary antibody (1:750; Abcam) at 16°C with constant shaking. The tissues were removed from the primary antibodies, washed three times (10 min each) with PBS and incubated in Goat anti-Rabbit Alexa Fluor 488 secondary antibody (Invitrogen) at RT for 1 h in the dark. Subsequently the tissues were washed three times (10 min each) with PBS and mounted with Prolong Anti-Fade mounting medium containing nuclear stain DAPI (Invitrogen). Care was taken not to let the tissue fold on itself during the mounting process.

### Imaging

Using an EVOS M7000 motorized-stage fluorescent microscope (Thermo Fisher), tissues were imaged with a 20× objective (EVOS AMEP4924; Fluorite LWD, 0.45 NA/6.23WD). Imaging was performed such that the entire width of the tissue was imaged over variable length. Care was taken not to image folded tissues. Initial concordance measurements of COUNTEN versus manual counting were done using images of individual fields. Subsequently, individual images were stitched together to generate a composite image that was used for COUNTEN analysis for generating the ENS map.

### Manual counting

Manual counting of HuC/D-immunostained neurons was performed by a trained technician using the plugin *Cell Counter* on ImageJ. Special attention was given to counting individual HuC/D labeled cells, without counting the same cell twice. Classification of neurons into ganglia was done by following the rule of defining a cluster of more than or equal to three neurons as a ganglion. Ganglionic boundaries were established as published in earlier studies (Kulkarni et al., 2017). The total number of ganglia per image and number of neurons per ganglia were thus enumerated and tabulated.

### Software

**Figure 1.**
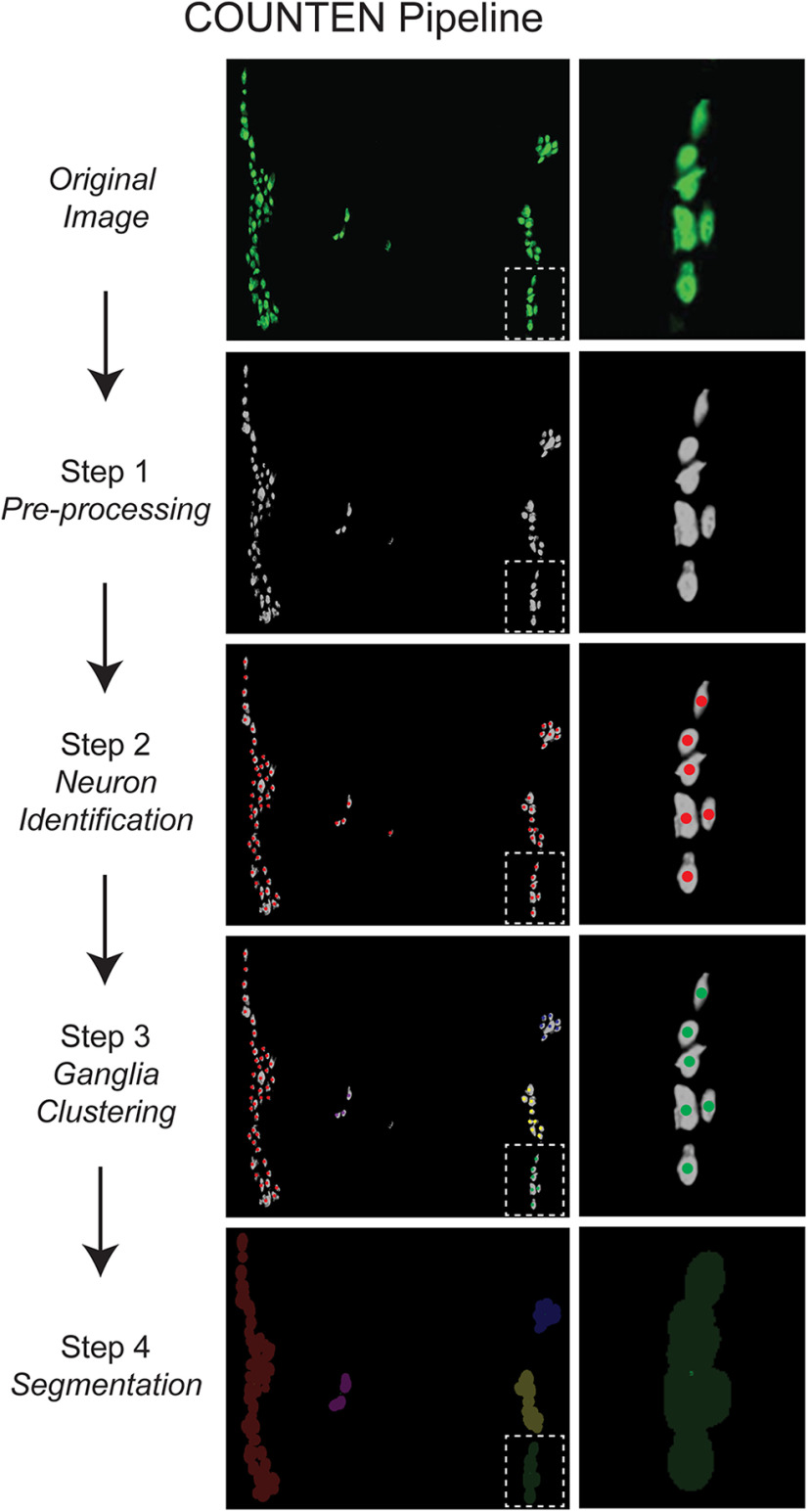
Representative images detailing the automatic COUNTEN image processing sequence for neuronal identification, enumeration, and clustering of HuC/D-immunostained iLM-MP tissue in a single 20× field of view. A single ganglion (in dotted box) is expanded on the right to show steps of neuronal identification, enumeration, and classification into ganglia.

The COUNTEN workflow consists of four sequential steps (Fig. 1): (1) image pre-processing; (2) neuron identification; (3) neuronal clustering into ganglia; and (4) image post-processing for segmentation. This algorithm was implemented in Python using the scikit-image, NumPy, and scikit-learn libraries. The same workflow was used for all the processing described in this report.

### Code accessibility

The COUNTEN software is freely available without restrictions on GitHub (https://github.com/KLab-JHU/COUNTEN) and is uploaded as a zip file with this submission (Extended Data 1).

10.1523/ENEURO.0092-21.2021.ed1 Extended Data 1 The COUNTEN software. Download Extended Data 1, ZIP file.

COUNTEN requires two user-specified parameters as input: the pixel density ρ (pixels/μm) as dictated by the imaging protocol, and the full width at half maximum σ (pixels) of a Gaussian smoothing kernel used during preprocessing. The four steps of the workflow are detailed below:

#### Image pre-processing

**Figure 2.**
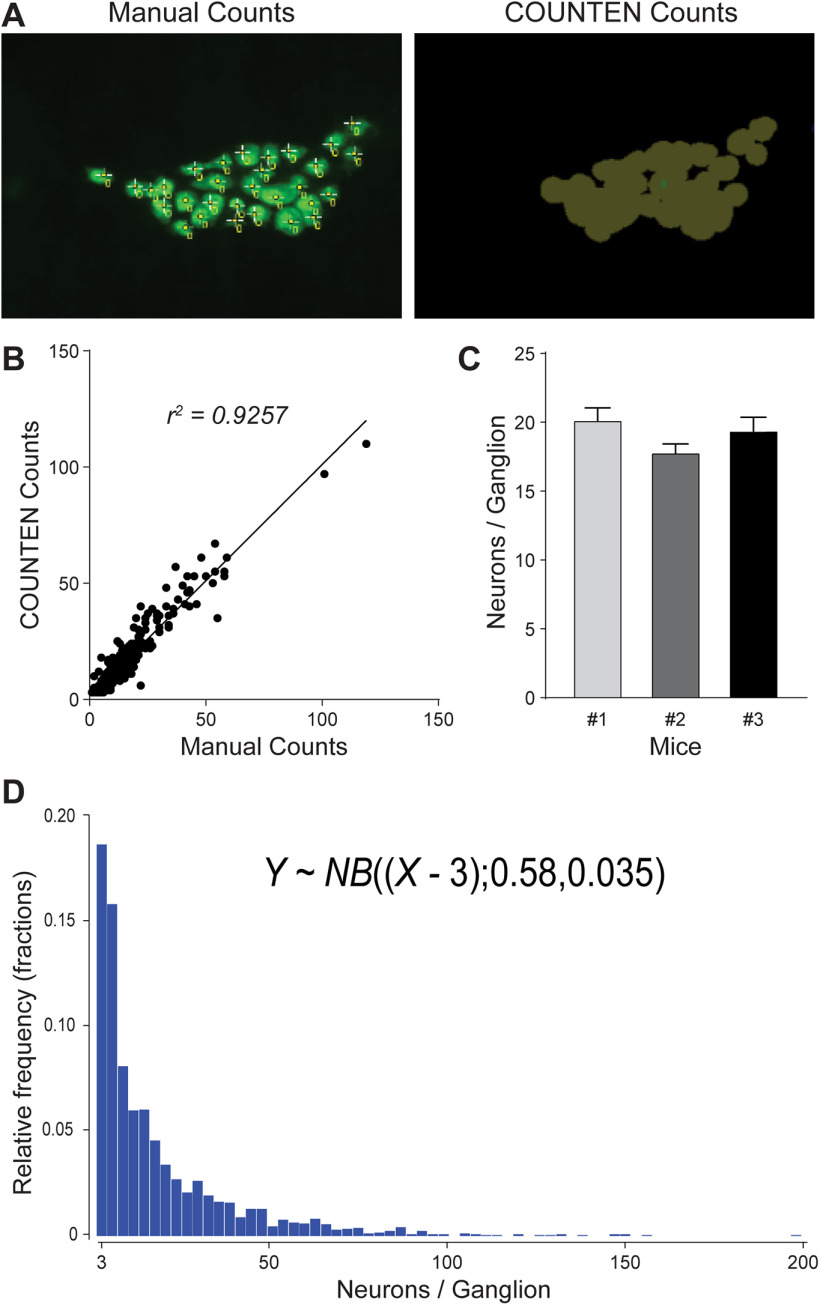
COUNTEN provides rapid, objective, and high-concordance identification, enumeration, and clustering of neurons. ***A***, Example of technician-driven “Manual” and “COUNTEN”-driven neuronal identification of the same myenteric ganglion. ***B***, High degree of correlation between COUNTEN-driven enumeration and technician-driven manual enumeration of myenteric neurons per ganglia from the same 100 20× HuC/D-immunostained images shows the conformity of COUNTEN and experienced technician-generated data. ***C***, COUNTEN-generated data of adult male iLM-MP tissue shows no significant difference in mean numbers of HuC/D-immunostained neurons/ganglia, suggesting a similar ganglia size between litter-mate male mice. Data are represented as mean ± SEM. ***D***, Frequency distribution histogram of ganglia size shows an inverse correlation between ganglia size and their relative abundance, as represented by the negative binomial equation. Values on the *x*-axis are in incremental bin sizes of three neurons per ganglion.

This step eliminates noise and staining variations, which might otherwise confound the results. We opted for a simple procedure, which can easily be replicated across different equipment configurations. The RGB image was first converted to a single grayscale channel and processed using an isotropic Gaussian filter. Larger blurs will reduce the contribution of extraganglionic neurons but also make the algorithm more susceptible to false negatives. Smaller blurs may result in insufficient de-noising of the image. We empirically determined that setting the Gaussian full width at half maximum to σ=7 pixels yields neuronal counts highly concordant with those of human raters (see Fig. 2). Hence, we fixed σ=7 for all analyses in this work. Next, we divided the image into nine equal partitions and used the center region to set a threshold between foreground (neurons) and background (GI tract). We use the center region to avoid biasing the threshold based on abnormalities at the tissue edges. The threshold is selected adaptively using Otsu’s method (Otsu, 1979), which minimizes the intraclass variance.

#### Neuron identification

This procedure searches for and returns all local maxima within the image, separated by a distance of at least $\delta_{m}$ (pixels). In other words, the peaks are local maxima of a circular neighborhood in the image with a prespecified radius of $\delta_{m}$. When there are multiple peaks within the same neighborhood, then the average of these coordinates is returned. We fixed the default value of $\delta_{m}$ according to the pixel density ρ as follows:

$$\delta_{m}\left(inpixels\right)=2.5\mu m\text{x }\rho .$$

We note that the parameter $\delta_{m}$ is accessible to users within the Python source code and can be modified from this default value as needed for other imaging protocols.

#### Clustering into ganglia

We used the density-based spatial clustering of applications with noise (DBSCAN) algorithm (Ester et al., 1996) to cluster the peak locations from step 2 into ganglia. DBSCAN is effective in our application since it does not assume a predefined number of clusters, and allows for unlabeled points (i.e., extraganglionic neurons). The DBSCAN algorithm takes as input two parameters, the minimum number of neurons in a ganglion $N_{g}$, and the minimum separation between ganglia $\varepsilon_{m}$. Here, we fixed $N_{g}=3$ (ganglia contain at least three neurons), and this convention was kept constant between COUNTEN-driven neuronal and ganglionic counts and manual counts by technician. We fixed the default value of $\varepsilon_{m}$ according to the pixel density ρ as follows:

$$\varepsilon_{m}\left(inpixels\right)=20.6\mu m\text{x }\rho .$$

Once again, the parameter $\varepsilon_{m}$ is accessible to users within the Python source code and can be modified from this default setting as needed.

#### Output segmentation

We binarized the image and used the watershed segmentation algorithm (Barnes et al., 2014) to flood the background pixels. This procedure leaves just the identified ganglia as the final output. The algorithm also colors the ganglia for ease of visualization.

## Results

We designed the COUNTEN algorithm to follow a sequence of four steps (Fig. 1): (1) image pre-processing using Otsu’s adaptive thresholding method (Otsu, 1979) to separate foreground neurons from background tissue; (2) neuron identification based on peak intensities within a local neighborhood; (3) neuronal clustering into ganglia using the DBSCAN algorithm (Ester et al., 1996); and (4) image post-processing via watershed segmentation (Barnes et al., 2014). We found that these four steps provide high-concordance data on the enumeration of neurons present in tissue and within defined ganglia (Fig. 1).

To test the concordance of COUNTEN over a larger number of images, we analyzed adult murine ileal LM-MP (iLM-MP) tissue. We first evaluated whether COUNTEN correctly identified and enumerated neurons with the same precision as human experts. We analyzed 100 images (*n* = 100), each representing a random 20× field of view of iLM-MP tissue immunostained with antibodies against pan-neuronal marker HuC/D. Each image depicted different numbers of neurons and we found that COUNTEN achieved high concordance with manual enumeration of neurons performed by human experts (Fig. 2*A*). In effect, every neuron identified by COUNTEN was also identified by manual counting.

Second, we evaluated the accuracy of the ganglionic clusters identified by COUNTEN. We defined a ganglion as a cluster of more than or equal to three neurons and defined ganglionic edges and boundaries by labeling the contours of the HuC/D-expressing cell clusters as previously published (Kulkarni et al., 2017). While manual counting identified 413 ganglia in total, COUNTEN achieved similar performance and identified 411 ganglia across the 100 images, underscoring COUNTEN’s reliability. Further, analysis of ganglia size exhibited a similar high degree of concordance between manual and COUNTEN methodologies (*r*^2^ = 0.9257; Y = 0.9998*X + 1.058; Fig. 2*B*). Beyond its reliability, COUNTEN offers a tremendous reduction in the time spent on the analysis. For reference, a technician took 2 d to analyze 100 images, while COUNTEN processed the same number of images in <10 min, providing us with a platform that performs rapid, precise, and objective neuronal and ganglionic counts in HuC/D-immunostained iLM-MP tissue.

Third, we used COUNTEN to quantify metrics that are often used for ganglionic organization within the iLM-MP. In contrast to our earlier tests of COUNTEN on single fields of views, here we deployed COUNTEN on widefield images generated from multiple stitched contiguous 20× images of HuC/D-immunostained iLM-MP tissue from three adult male littermate mice. We imaged areas of 46.15, 48.83, and 36.34 mm^2^, which we found to contain 15741, 13268, and 9247 neurons within 778, 742, and 475 ganglia, respectively. Using COUNTEN-generated data, we calculated the neuronal density in the three tissues to be 344.33, 288.21, and 269.03 neurons/mm^2^ and the ganglionic density was calculated to be 16.86, 15.19, 13.07 ganglia/mm^2^.

Finally, we used COUNTEN-generated data to study mean ganglia size and ganglia diversity. Ganglia size in none of the three tissues followed normal distribution (Anderson Darling test, *p* < 0.0001), and the mean ganglia size between the three tissues, computed to be 20.23, 17.88 and 19.47 neurons/ganglia, were not statistically different (Kruskal–Wallis test; Fig. 2*C*). The frequency distribution of ganglia size across the three tissues showed inverse correlation of ganglia size and their relative abundance (Fig. 2*D*), which can be summarized by a negative binomial equation:

Y ∼ NB((X−3);0.58,0.035).

## Discussion

The ENS has been previously described at an anatomic, physiological, and transcriptomic level (Costa et al., 2000; Spencer and Hu, 2020; Morarach et al., 2021), yet a detailed, overarching structural analysis of ENS plexuses within defined intestinal tissue areas has not been performed. This can be attributed to the lack, until now, of reliable tools and methods for enumerating large numbers of enteric neurons from distinct tissue segments and clustering them into ganglia.

In this report, we describe the generation and methodology for use of COUNTEN, the first open-source tool for rapid, automated and objective enumeration and clustering of ENS neurons. Prior tools such as ImageJ, while providing modules and macros that allow for correct enumeration of neurons, require human intervention to identify and cluster neurons into ganglia, making the procedure slow and subjective (Keating et al., 2013). COUNTEN, on the other hand, relies solely on computer-vision algorithms to parse a large number of images in a short duration of time, and applies a single definition for neuronal clustering equally to all the images to produce rapid and objective neuronal and ganglionic enumeration.

To showcase the ability of COUNTEN, we compared human manual counting using an already available tool, ImageJ, with COUNTEN to analyze 100 images of immunostained-iLM-MP tissue. While COUNTEN took a fraction of the time that a trained technician took to parse through the images, we found there to be a high degree of conformity between manual counting and COUNTEN-generated data (Fig. 2*B*), suggesting that the gains in speed with COUNTEN were not associated with a loss in accuracy with regards to the identification and enumeration of neurons and their classification into ganglia.

The rapidity with which COUNTEN analyzes immunostained images of iLM-MP tissue allowed us to generate high-fidelity data on neuronal numbers and ganglia size from a large region of tissue. We analyzed these data to show how COUNTEN can help the scientific community construct large scale statistical maps of the ENS. We used COUNTEN-generated data to establish that ganglia size is conserved across ileal tissues from sex-matched littermate mice. We further used these data to establish the parameters of a negative binomial equation that defines the frequency distribution of ileal ganglia size. Along with neuronal and ganglionic density, this equation provides the metrics of ganglionic organization in the iLM-MP and can be used as a reference for future studies. These analyses show how rapid and objective neuronal enumeration by COUNTEN can help the scientific community define ENS structure in animal models of health and disease.

While COUNTEN is a significant advance in our ability to interrogate ENS structure, it is a first-generation tool and has a few limitations. The performance of COUNTEN depends on the robustness and homogeneity of immunostaining and imaging, which we have standardized (see Materials and Methods). Further, while ganglia are three-dimensional (3D) structures, the current algorithm does not operate on 3D image-data. However, the high concordance between COUNTEN and human-generated data, suggests that the data generated by COUNTEN are as accurate as those generated by a human observer. Finally, COUNTEN is currently limited to interrogating HuC/D-immunolabeled neurons and does not have the ability to query two different immunolabels. However, to facilitate rapid and reproducible measurement of ENS structure within the broader ENS community and to propel the development of this tool in an open-source manner, COUNTEN is freely and openly available to researchers. As an open-source, freely-available tool under active development, the authors and the scientific community alike can add modules to the existing program that may increase the future functionality of COUNTEN.

## Synthesis

Reviewing Editor: Karen Davis, Krembil Research Institute and University of Toronto Decisions are customarily a result of the Reviewing Editor and the peer reviewers coming together and discussing their recommendations until a consensus is reached. When revisions are invited, a fact-based synthesis statement explaining their decision and outlining what is needed to prepare a revision will be listed below. The following reviewer(s) agreed to reveal their identity: Nick Spencer.

This study demonstrates a new analytical platform that performs rapid, precise, and objective neuronal and ganglionic counts of immunostained neurons in the Enteric Nervous System (ENS). This is a great new tool for neuroscience, especially the ENS. There is a strong demand for a tool like this software approach. And, I certainly agree with the author's statement “..there is a critical need for objective and rapid tools and methods for standardized enumeration and classification of myenteric neurons into ganglia over large tissue..”

I commend the author's for their study. This analytical approach is shown to be accurate and tremendously time-saving. Great ! The authors used COUNTEN-generated data to establish that ganglia size is conserved across ileal tissues from sex-matched littermate mice. I do have one resolvable, but important concern regarding the decay equation. A couple of extra references also need to be inserted. Overall, great work ! With corrections this paper, will be a very useful tool for science.

MAJOR:

My only major concern (which can be dealt with), is that I am not sure the decay equation correctly models ganglia size distribution. A distribution of ganglia size should integrate to 100%, but the given equation is unbounded. For example, integrating ganglia sizes between 100 neurons/ganglia and 1000 neurons/ganglia results in 108%, suggesting 108% of all ganglia have sizes between 100 and 1000. Even over the 3..200 domain given in figure 2D, integrating the equation results in 245% of ganglia having neuron counts between 3 and 200. This is clearly an untenable result. I suggest the authors instead fit a Poisson or negative binomial probability distribution to the neuron counts, rather than fitting an arbitrary curve.

Other corrections required:

One page 3, the sentence “...Animal Care and Use Committee of [Author University] in..” please insert the University and delete “Author University"

Page 8, last paragraph: the sentence I am not sure the authors chose the right word here (parses). “The rapidity with which COUNTEN parses through immunostained...” Didn't the author's mean to say: “The rapidity with which COUNTEN passes through immunostained...”

At the end of the first sentence of the introduction insert reference to Spencer NJ & Hu H (2020) reference below. Also insert this reference at the end of the first sentence of the Discussion.

Under the heading “Tissue isolation"

Delete: “were” between “..and were sacrificed..”

Page 7, line 5: The sentence needs a reference. This is incomplete: “...previously published (REF).”

Page 8, line 11: at the end of the sentence ending with “...procedure slow and subjective.” Insert reference to Keating DJ et al. (2013) Neurogastroenterology & Motility, 25(10) 849053. https://pubmed.ncbi.nlm.nih.gov/23901879/

References that need to be inserted.

Spencer NJ & Hu H (2020) The Enteric Nervous System. Sensory transduction, neural circuits and gastrointestinal motility. Nat Rev Gastroenterol Hepatol. 2020 Mar 9. doi: 10.1038/s41575-020-0271-2.

https://pubmed.ncbi.nlm.nih.gov/32152479/

Keating DJ et al. (2013) Neurogastroenterology & Motility, 25(10) 849053. https://pubmed.ncbi.nlm.nih.gov/23901879/

## Author Response

Response to reviewers:

We are submitting point-by-point responses to the reviewers comments and suggested edits.

Synthesis Statement for Author (Required):

This study demonstrates a new analytical platform that performs rapid, precise, and objective neuronal and ganglionic counts of immunostained neurons in the Enteric Nervous System (ENS). This is a great new tool for neuroscience, especially the ENS.

There is a strong demand for a tool like this software approach. And, I certainly agree with the author's statement “..there is a critical need for objective and rapid tools and methods for standardized enumeration and classification of myenteric neurons into ganglia over large tissue..”

We thank the reviewer for considering our work significant and useful.

I commend the author's for their study. This analytical approach is shown to be accurate and tremendously time-saving. Great ! The authors used COUNTEN-generated data to establish that ganglia size is conserved across ileal tissues from sex-matched littermate mice. I do have one resolvable, but important concern regarding the decay equation. A couple of extra references also need to be inserted. Overall, great work ! With corrections this paper, will be a very useful tool for science.

We thank the reviewer for their review.

My only major concern (which can be dealt with), is that I am not sure the decay equation correctly models ganglia size distribution. A distribution of ganglia size should integrate to 100%, but the given equation is unbounded. For example, integrating ganglia sizes between 100 neurons/ganglia and 1000 neurons/ganglia results in 108%, suggesting 108% of all ganglia have sizes between 100 and 1000. Even over the 3..200 domain given in figure 2D, integrating the equation results in 245% of ganglia having neuron counts between 3 and 200. This is clearly an untenable result. I suggest the authors instead fit a Poisson or negative binomial probability distribution to the neuron counts, rather than fitting an arbitrary curve.

We thank the reviewer for their comment. We certainly agree with their assessment of the unsuitability of the One-Step Decay equation and also agree that alternate distribution equations should be considered. We plotted our data and found that the negative binomial distribution fits our observed data best and have suitably amended the manuscript and figures to show the distribution and the negative binomial equation that models the distribution.

One page 3, the sentence “...Animal Care and Use Committee of [Author University] in..” please insert the University and delete “Author University"

To ensure double blinded reviews, the journal has specifically asked us not to provide any identifying information in the resubmission.2 Page 8, last paragraph: the sentence I am not sure the authors chose the right word here (parses). “The rapidity with which COUNTEN parses through immunostained...” Didn't the author's mean to say: “The rapidity with which COUNTEN passes through immunostained...”

This has been amended in the revised resubmitted manuscript. The sentence now reads “The rapidity with which COUNTEN analyzes immunostained....”

At the end of the first sentence of the introduction insert reference to Spencer NJ & Hu H (2020) reference below. Also insert this reference at the end of the first sentence of the Discussion.

We thank the reviewers for suggesting this reference and this has been added to the revised resubmitted manuscript.

Under the heading “Tissue isolation" Delete: “were” between “..and were sacrificed..”

This has been amended in the revised resubmitted manuscript. Page 7, line 5: The sentence needs a reference. This is incomplete: “...previously published (REF).”

The missing reference has been added to the revised resubmitted manuscript.

Page 8, line 11: at the end of the sentence ending with “...procedure slow and subjective.” Insert reference to Keating DJ et al. (2013) Neurogastroenterology & Motility, 25(10) 849053.

We thank the reviewers for suggesting this reference and this has been added to the revised resubmitted manuscript

## References

[B1] Barnes R, Lehman C, Mulla D (2014) Priority-flood: an optimal depression-filling and watershed-labeling algorithm for digital elevation models. Comput Geosci 62:117–127. 10.1016/j.cageo.2013.04.024

[B2] Becker L, Nguyen L, Gill J, Kulkarni S, Pasricha PJ, Habtezion A (2018) Age-dependent shift in macrophage polarisation causes inflammation-mediated degeneration of enteric nervous system. Gut 67:827–836. 10.1136/gutjnl-2016-312940 28228489 PMC5565713

[B3] Costa M, Brookes SJ, Hennig GW (2000) Anatomy and physiology of the enteric nervous system. Gut 47:iv15–19. 10.1136/gut.47.suppl_4.iv15 11076898 PMC1766806

[B4] Ester M, Kriegel H-P, Sander J, Xu X (1996) A density-based algorithm for discovering clusters in large spatial databases with noise. Proceedings of the Second International Conference on Knowledge Discovery and Data Mining, pp 226–231. Portland: AAAI Press.

[B5] Gabella G (1987) The number of neurons in the small intestine of mice, guinea-pigs and sheep. Neuroscience 22:737–752. 10.1016/0306-4522(87)90369-1 2444903

[B6] Gianino S, Grider JR, Cresswell J, Enomoto H, Heuckeroth RO (2003) GDNF availability determines enteric neuron number by controlling precursor proliferation. Development 130:2187–2198. 10.1242/dev.00433 12668632

[B7] Keating DJ, Peiris H, Kyloh M, Brookes SJ, Spencer NJ (2013) The presence of 5-HT in myenteric varicosities is not due to uptake of 5-HT released from the mucosa during dissection: use of a novel method for quantifying 5-HT immunoreactivity in myenteric ganglia. Neurogastroenterol Motil 25:849–853. 23901879 10.1111/nmo.12189

[B8] Kulkarni S, Micci MA, Leser J, Shin C, Tang SC, Fu YY, Liu L, Li Q, Saha M, Li C, Enikolopov G, Becker L, Rakhilin N, Anderson M, Shen X, Dong X, Butte MJ, Song H, Southard-Smith EM, Kapur RP, et al. (2017) Adult enteric nervous system in health is maintained by a dynamic balance between neuronal apoptosis and neurogenesis. Proc Natl Acad Sci USA 114:E3709–E3718. 10.1073/pnas.1619406114 28420791 PMC5422809

[B9] Kunze WAA, Furness JB (1999) The enteric nervous system and regulation of intestinal motility. Annu Rev Physiol 61:117–142. 10.1146/annurev.physiol.61.1.117 10099684

[B10] Morarach K, Mikhailova A, Knoflach V, Memic F, Kumar R, Li W, Ernfors P, Marklund U (2021) Diversification of molecularly defined myenteric neuron classes revealed by single-cell RNA sequencing. Nat Neurosci 24:34–46. 10.1038/s41593-020-00736-x 33288908 PMC7610403

[B11] Otsu N (1979) A threshold selection method from gray-level histograms. IEEE Trans Syst Man Cybern 9:62–66. 10.1109/TSMC.1979.4310076

[B12] Rao M, Gershon MD (2018) Enteric nervous system development: what could possibly go wrong? Nat Rev Neurosci 19:552–565. 10.1038/s41583-018-0041-0 30046054 PMC6261281

[B13] Santer RM, Baker DM (1988) Enteric neuron numbers and sizes in Auerbach’s plexus in the small and large intestine of adult and aged rats. J Auton Nerv Syst 25:59–67. 10.1016/0165-1838(88)90008-2 3225382

[B14] Spencer NJ, Hu H (2020) Enteric nervous system: sensory transduction, neural circuits and gastrointestinal motility. Nat Rev Gastroenterol Hepatol 17:338–351. 10.1038/s41575-020-0271-2 32152479 PMC7474470

[B15] Sternini C (1988) Structural and chemical organization of the myenteric plexus. Annu Rev Physiol 50:81–93. 10.1146/annurev.ph.50.030188.000501 3288110

